# Assessment of type I interferon signatures in undifferentiated inflammatory diseases: A Japanese multicenter experience

**DOI:** 10.3389/fimmu.2022.905960

**Published:** 2022-09-23

**Authors:** Takayuki Miyamoto, Yoshitaka Honda, Kazushi Izawa, Nobuo Kanazawa, Saori Kadowaki, Hidenori Ohnishi, Masakazu Fujimoto, Naotomo Kambe, Naoya Kase, Takeshi Shiba, Yasuo Nakagishi, Shuji Akizuki, Kosaku Murakami, Masahiro Bamba, Yutaka Nishida, Ayano Inui, Tomoo Fujisawa, Daisuke Nishida, Naomi Iwata, Yoshikazu Otsubo, Shingo Ishimori, Momoko Nishikori, Kiminobu Tanizawa, Tomoyuki Nakamura, Takeshi Ueda, Yoko Ohwada, Yu Tsuyusaki, Masaki Shimizu, Takasuke Ebato, Kousho Iwao, Akiharu Kubo, Toshinao Kawai, Tadashi Matsubayashi, Tatsuhiko Miyazaki, Tomohiro Kanayama, Masahiko Nishitani-Isa, Hiroshi Nihira, Junya Abe, Takayuki Tanaka, Eitaro Hiejima, Satoshi Okada, Osamu Ohara, Megumu K. Saito, Junko Takita, Ryuta Nishikomori, Takahiro Yasumi

**Affiliations:** ^1^ Department of Pediatrics, Kyoto University Graduate School of Medicine, Kyoto, Japan; ^2^ Institute for the Advanced Study of Human Biology (ASHBi), Kyoto University, Kyoto, Japan; ^3^ Department of Immunology, Kyoto University Graduate School of Medicine, Kyoto, Japan; ^4^ Department of Dermatology, Hyogo Medical University, Nishinomiya, Japan; ^5^ Department of Pediatrics, Gifu University Graduate School of Medicine, Gifu, Japan; ^6^ Department of Diagnostic Pathology, Kyoto University Hospital, Kyoto, Japan; ^7^ Department of Dermatology, Kyoto University Graduate School of Medicine, Kyoto, Japan; ^8^ Department of Clinical Application, Center for iPS cell (Induced pluripotent stem cell) Research and Application, Kyoto University, Kyoto, Japan; ^9^ Department of Pediatrics, Tenri Hospital, Tenri, Japan; ^10^ Department of Pediatric Rheumatology, Hyogo Prefectural Kobe Children’s Hospital, Kobe, Japan; ^11^ Division of Clinical Immunology and Cancer Immunotherapy, Center for Cancer Immunotherapy and Immunobiology, Graduate School of Medicine, Kyoto University, Kyoto, Japan; ^12^ Department of Pediatrics, Kawasaki Municipal Hospital, Kawasaki, Japan; ^13^ Department of Pediatrics, Gunma University Graduate School of Medicine, Maebashi, Japan; ^14^ Department of Pediatric Hepatology and Gastroenterology, Saiseikai Yokohamashi Tobu Hospital, Yokohama, Japan; ^15^ Department of Infection and Immunology, Aichi Children’s Health and Medical Center, Aichi, Japan; ^16^ Department of Pediatrics, Sasebo City General Hospital, Sasebo, Japan; ^17^ Department of Pediatrics, Takatsuki General Hospital, Takatsuki, Japan; ^18^ Department of Hematology and Oncology, Graduate School of Medicine, Kyoto University, Kyoto, Japan; ^19^ Department of Respiratory Medicine, Graduate School of Medicine, Kyoto University, Kyoto, Japan; ^20^ Department of General Medicine, Osaka City Hospital Organization Osaka City General Hospital, Osaka, Japan; ^21^ Department of Emergency and General Internal Medicine, Rakuwakai Marutamachi Hospital, Kyoto, Japan; ^22^ Department of Pediatrics, Dokkyo Medical University School of Medicine, Tochigi, Japan; ^23^ Department of Neurology, Kanagawa Children’s Medical Center, Yokohama, Japan; ^24^ Department of Pediatrics and Developmental Biology, Graduate School of Medical and Dental Sciences, Tokyo Medical and Dental University, Tokyo, Japan; ^25^ Department of Pediatrics, Kitasato University, School of Medicine, Kanagawa, Japan; ^26^ Department of Internal Medicine, Division of Rheumatology, Infectious Diseases and Laboratory Medicine, University of Miyazaki, Miyazaki, Japan; ^27^ Department of Dermatology, Keio University School of Medicine, Tokyo, Japan; ^28^ Division of Immunology, National Center for Child Health and Development, Tokyo, Japan; ^29^ Department of Pediatrics, Seirei Hamamatsu General Hospital, Hamamatsu, Japan; ^30^ Department of Pathology, Gifu University Hospital, Gifu, Japan; ^31^ Department of Pediatrics, Kitano Hospital, Tazuke Kofukai Medical Research Institute, Osaka, Japan; ^32^ Department of Pediatrics, Otsu Red Cross Hospital, Otsu, Japan; ^33^ Department of Pediatrics, Hiroshima University Graduate School of Biomedical and Health Sciences, Hiroshima, Japan; ^34^ Department of Applied Genomics, Kazusa DNA Research Institute, Kisarazu, Japan; ^35^ Department of Pediatrics and Child Health, Kurume University School of Medicine, Kurume, Japan

**Keywords:** interferon, interferon signature, interferonopathy, autoinflammation, A20 haploinsufficiency, pulmonary hemosiderosis

## Abstract

**Purpose:**

Upregulation of type I interferon (IFN) signaling has been increasingly detected in inflammatory diseases. Recently, upregulation of the IFN signature has been suggested as a potential biomarker of IFN-driven inflammatory diseases. Yet, it remains unclear to what extent type I IFN is involved in the pathogenesis of undifferentiated inflammatory diseases. This study aimed to quantify the type I IFN signature in clinically undiagnosed patients and assess clinical characteristics in those with a high IFN signature.

**Methods:**

The type I IFN signature was measured in patients’ whole blood cells. Clinical and biological data were collected retrospectively, and an intensive genetic analysis was performed in undiagnosed patients with a high IFN signature.

**Results:**

A total of 117 samples from 94 patients with inflammatory diseases, including 37 undiagnosed cases, were analyzed. Increased IFN signaling was observed in 19 undiagnosed patients, with 10 exhibiting clinical features commonly found in type I interferonopathies. Skin manifestations, observed in eight patients, were macroscopically and histologically similar to those found in proteasome-associated autoinflammatory syndrome. Genetic analysis identified novel mutations in the *PSMB8* gene of one patient, and rare variants of unknown significance in genes linked to type I IFN signaling in four patients. A JAK inhibitor effectively treated the patient with the *PSMB8* mutations. Patients with clinically quiescent idiopathic pulmonary hemosiderosis and A20 haploinsufficiency showed enhanced IFN signaling.

**Conclusions:**

Half of the patients examined in this study, with undifferentiated inflammatory diseases, clinically quiescent A20 haploinsufficiency, or idiopathic pulmonary hemosiderosis, had an elevated type I IFN signature.

## Introduction

A critical role for type I interferons (IFNs) in the pathogenesis of inflammatory diseases has been increasingly recognized in recent years. IFNs are a group of cytokines that play an important role in host defense against viruses. IFNs consist of three distinct families, namely, type I (IFNα/β/ϵ/τ/κ/ω/δ/ζ), II (IFNγ), and III (IFNλ).IFNα and IFNβ are the most understood, and broadly expressed type I IFNs. They are produced by most cell types in response to stimulation from pattern recognition receptors through intracellular and endosomal nucleic acids. Once secreted extracellularly, they bind to type I IFN receptors and activate hundreds of IFN stimulated genes (ISGs), which affect the innate and adaptive immune response (1, 2).

In 2003, multiple investigators reported that peripheral blood cells from systemic lupus erythematosus (SLE) patients demonstrated an overexpression of a characteristic pattern of ISGs, termed an IFN signature (3–5). Although there is a large overlap between the ISGs induced by all three IFN families, the primary IFN signature is most consistent with induction from type I IFNs (6, 7). Since detection of type I IFN in human serum by conventional enzyme-linked immunosorbent assay (ELISA) is complicated by low reproducibility and poor correlation with functional assay (8), expression of an IFN signature has been widely used to assess type I IFN activity. An increased IFN signature has been identified in many autoimmune diseases, including SLE, rheumatic arthritis (RA), systemic sclerosis (SSc) and dermatomyositis (DM), and its utility as a biomarker to predict disease severity or to assess disease activity is readily studied (9–16).

Type I IFNs are also involved in the pathogenesis of autoinflammatory diseases. In 2011, Crow et al. proposed the concept of type I interferonopathy, which refers to a group of Mendelian inflammatory disorders where chronic and autonomous enhancement of type I IFN production was posited as directly relevant to pathogenesis (17). Since then, numerous Mendelian genotypes were found to be associated with enhanced type I IFN signaling (18). Several investigators have suggested the utility of a type I IFN signature as a biomarker to distinguish patients with type I interferonopathy from those with other inflammatory diseases (19–22). Moreover, several groups reported that a type I IFN signature correlates with disease activity and treatment from JAK inhibitors, suggesting that an IFN signature may serve as a biomarker to monitor treatment response (23).

Recent studies have examined the efficacy of JAK inhibitors for the treatment of various autoimmune and autoinflammatory diseases (23–29), suggesting a causal relationship between enhanced type I IFN signaling and disease pathogenesis. Clinically, it is becoming more important to diagnose type I IFN-driven inflammatory diseases rapidly and accurately for personalized medical treatment. In this report, the expression of a type I IFN signature in patients with various inflammatory diseases was investigated. An increased IFN signature was detected in disease states, whose etiologies have not previously been associated with type I IFNs, and in some patients with clinically and genetically undiagnosed inflammatory diseases. In some patients where enhanced IFN signaling was observed, a retrospective assessment of clinical phenotypes revealed characteristics similar to monogenic, type I interferonopathies. In particular, skin manifestations in these cases were macroscopically and histologically similar. These findings may be indicative of a pathogenetic role for type I IFNs in these diseases and suggest the existence of unknown genotypes, which may lead to the upregulation of type I IFN signaling.

## Material and methods

### Patients and healthy controls

Patients, suspected of having autoinflammatory or undifferentiated autoimmune diseases, were selected for this study based on the recommendation of their attending physician. Samples were collected from a total of 117 individuals, comprising 57 patients with diagnosed inflammatory diseases and 37 patients with undifferentiated inflammatory diseases who had no genetic or clinical diagnosis upon referral. Eleven Japanese adults who self-reported to have no known medical conditions or infection symptoms were recruited as healthy controls (HCs). Asymptomatic pediatric patients with noninflammatory diseases, such as congenital heart disease and hydronephrosis, who attended a hospital for a routine examination were recruited as the pediatric controls. IFN signatures were measured in 140 samples collected between 2016 and 2020 (**Table S1**).

### Study approval

The ethics committee of Kyoto University approved this study, which was conducted in accordance with the Helsinki Declaration. Written informed consent was obtained from all of the subjects or legally authorized representative

### Clinical and genetic evaluation

Clinical and biological data from the 37 patients with undifferentiated inflammatory diseases was collected retrospectively from their medical records or from interviews with the attending physician.

An in-depth genetic analysis was performed on all 19 patients with undifferentiated inflammatory diseases whose type I IFN signature was elevated. Trio-based, whole exome sequencing (WES) was conducted on ten of the patient samples, while targeted genomic sequencing (TS), that analyzed a panel of 533 genes associated with immunodeficiency and autoinflammatory diseases, including monogenic type I interferonopathies, was completed on the other nine patient samples.

### IFN score (IS)

The IFN signature was measured using quantitative reverse transcription polymerase chain reaction (RT-qPCR) as described previously (19). Briefly, total RNA was extracted from whole blood using the PAXgene Blood RNA kit (PreAnalytix, Hombrechtikon, Switzerland). Gene expression of six ISGs (IFI27, IFI44L, IFIT1, ISG15, RSAD2, and SIGLEC1) was then determined by RT-qPCR using cDNA derived from 40 ng of total RNA and the TaqMan™ Gene Expression Master Mix (Thermo Fisher Scientific, Waltham, MA). PCR was performed using the StepOnePlus™ Real-Time PCR System (Thermo Fisher Scientific). TaqMan probes for IFI27 (Hs01086370_m1), IFI44L (Hs00199115_m1), IFIT1 (Hs00356631_g1), ISG15 (Hs00192713_m1), RSAD2 (Hs01057264_m1), SIGLEC1 (Hs00988063_m1), and β actin (HS01060665_g1) were used. The relative abundance of each target gene transcript was measured using the ΔΔCT method. The expression of each ISG in each patient was normalized to the β actin expression level and then calculated relative to the median expression level of the 11 HCs. The IS was defined as the median relative expression level of the six ISGs. An abnormal IS was defined as that greater than two standard deviations from the mean IS in the control group (i.e., 5.04).

### Proteasome activity detection

For the detection of chymotrypsin activity, monocytes isolated using the autoMACS® cell separator (Miltenyi Biotec, Bergisch Gladbach, Germany) from peripheral blood mononuclear cells, obtained by Lymphoprep™ (STEMCELL Technologies, Vancouver, Canada) separation, were seeded at a concentration of 1 × 104 cells/well in a white 96-well plate. DMSO or 1 μM ONX-0914 (Adooq Bioscience, Irvine, CA) was added, and 3 hours later, TNF-α and IFN-γ (100 ng/mL) were added to stimulate the cells. After culturing the cells for 21 hours, 100 μL Proteasome-Glo™ Chymotrypsin-Like Cell-Based Assay (Promega, Madison, WI) was added for 1 hour at 37°C. Then, chemiluminescence was detected using the 2104 EnVision Multilabel Plate Reader (PerkinElmer, Waltham, MA).

To detect β5 and β5i subunit activity, 3 × 104 cells were seeded into each well of white 96-well plates. DMSO or 1 μM ONX-0914 was added to the wells, and 3 hours later, the cells were stimulated with TNF-α and IFN-γ (100 ng/mL). After culturing the cells for 21 hours, Ac-Trp-Leu-Ala-AMC (R&D Systems, Inc., Minneapolis, MN) to detect β5 activity, or Ac-Ala-Asn-Trp-AMC (R&D Systems, Inc.) to detect β5i activity, was added to a final concentration of 50 μM, followed by incubation at 37°C for the indicated times (**Figure 3B**). AMC fluorescence was detected using the 2104 EnVision Multilabel Plate Reader.

### Immunohistochemistry

Tissue sections (4 µm thick) from archived paraffin-embedded tissue blocks were prepared for immunohistochemical and hematoxylin and eosin (H&E) staining. Immunohistochemistry was performed using antibodies against CD3 (2GV6; Roche, Basel, Switzerland), CD20 (L26; DAKO, Santa Clara, CA), CD15 (Carb-3; DAKO), CD123 (6H6; Thermo Fischer Scientific), CD163 (10D6; Leica Microsystems, Wetzlar, Germany), or MPO (polyclonal; DAKO). All staining procedures were performed using an autoimmunostainer (Bond III [Leica Microsystems] or BenchMark Ultra [Ventana Medical Systems, Oro Valley, AZ]).

### Statistical analysis

Descriptive statistical analyses were performed and differences in proportions between the groups in **Table 1** were evaluated by a Fisher’s exact test. Results for **Figure S2** were analyzed using a one-way ANOVA with a Dunnett’s multiple comparisons test. All statistical analyses, described above, were performed using the GraphPad Prism software version 8.00 (GraphPad Software, La Jolla, California, USA, www.graphpad.com).

**Table 1:** Comparison of clinical phenotypes in patients with and without enhanced IFN signaling.

	Patients with high IS Number of patientsaffected/evaluated (%)(n=19)		Patients without high IS Number of patientsaffected/evaluated (%)(n=18)		Fisher’s exact test p-value a
**Age at onset** **(median [IQ range]), months**	8	(1–96)	180	(78-286)	0.007
**Male**	7/19	(36.8)	6/13	(35.3)	>0.99
**Fever**	14/19	(73.7)	14/18	(77.8)	>0.99
**Skin involvement**	14/19	(73.7)	10/18	(55.6)	0.31
**- chilblain**	6/19	(31.6)	0/18	(0)	0.02
**- nodular erythema**	9/19	(47.4)	3/18	(16.7)	0.08
**Panniculitis**	2/19	(10.5)	0/18	(0)	0.49
**Myositis**	2/19	(10.5)	0/18	(0)	0.49
**Arthritis/Arthralgia**	4/19	(21.0)	3/18	(16.7)	>0.99
**Interstitial pneumoniae**	1/19	(5.3)	0/18	(0)	>0.99
**CNS manifestation**					
**- headache**	2/19	(10.5)	4/18	(22.2)	0.40
**- intracranial calcification**	1/14	(7.1)	0/7	(0)	>0.99
**- aseptic meningitis**	0/19	(0)	1/18	(5.6)	0.49
**Transaminitis**	7/19	(36.8)	1/18	(5.6)	0.042
**Autoantibody**	7/19	(36.8)	2/17	(11.8)	0.13
**- including ANA (1:40 and 1:80)**	13/19	(68.4)	3/17	(17.6)	0.003

a; Difference in age at onset was analyzed using a Mann-Whitney test. Two patients from each group whose age of disease onset was ambiguous were excluded from the statistical analysis for this category. All four of these patients self-reported that they had symptoms since early childhood. ANA, antinuclear antibody.

## Results

### The type I IFN signature in clinically or genetically defined cases

**Figure 1:**
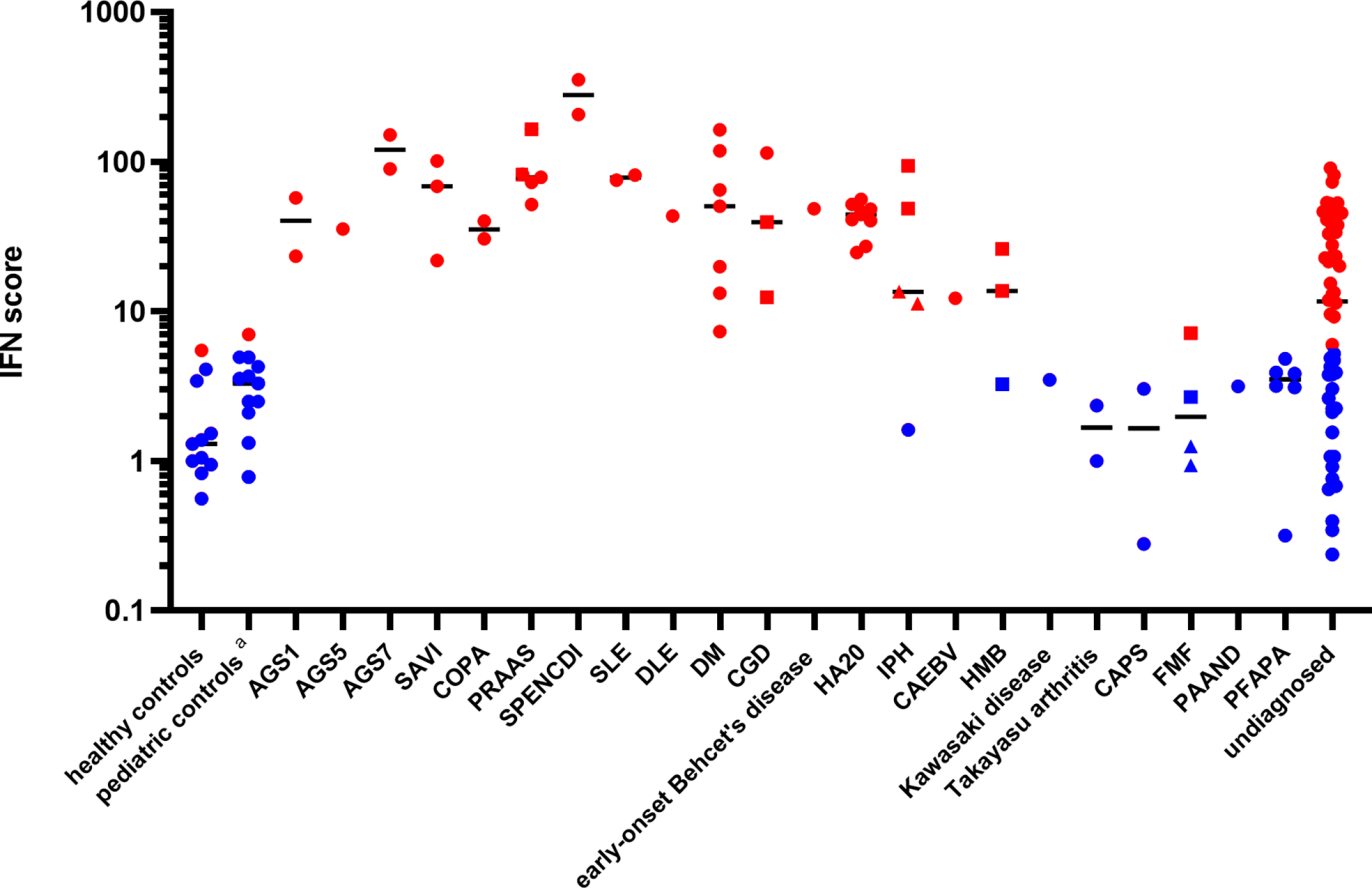
Patient interferon scores according to disease diagnosis. Red dots represent ISs greater than 5.04, while blue dots represent ISs below 5.04, based on two standard deviations from the mean score found in healthy controls. The circles represent the ISs of the different subjects, except for those of the “undiagnosed” patients with a high IS. The squares and triangles represent repeat samples from the same subjects, respectively. Details of the ISs of the “undiagnosed” patients with a high IS are described in **Table 2**. Black horizontal lines represent the median for each patient group. DLE, discoid lupus erythematosus; CAEBV, chronic active Epstein-Barr virus infection; HMB, hypersensitivity to mosquito bites; FMF, familial Mediterranean fever; PAAND, pyrin-associated autoinflammation with neutrophilic dermatosis; PFAPA, periodic fever, aphthous stomatitis, pharyngitis, cervical adenitis. The pediatric controls include asymptomatic pediatric patients with noninflammatory diseases such as congenital heart disease and hydronephrosis who attended a hospital for a routine examination.

Patients ISs were plotted, according to disease diagnosis, as shown in **Figure 1**. The relative expression levels of each ISG are presented as a heatmap in Figure S1. All patients with monogenic type I interferonopathies, including Aicardi-Goutières syndrome (AGS); proteasome-associated autoinflammatory syndrome (PRAAS); STING-associated vasculopathy with onset in infancy (SAVI); COPA syndrome; spondyloenchondrodysplasia with immune dysregulation (SPENCDI); and other monogenic and polygenic diseases, which are associated with the upregulation of type I IFN signaling, including chronic granulomatous disease (CGD); SLE; and DM; demonstrated high ISs.

A20 haploinsufficiency (HA20) is a systemic autoinflammatory disease caused by a heterozygous loss-of-function mutation in the TNF-α-induced protein 3 (TNFAIP3). Recent reports have demonstrated the elevation of a type I IFN signature and potential therapeutic benefits of JAK inhibitors in HA20 patients (30, 31). Since nine patients (from six unrelated families) in the study were diagnosed with HA20, their type I interferon signature was analyzed. Determination of the patients’ genotypes and TNFAIP3 variants revealed several previously reported variants (32) and as well as two variants (p.Lys329Asn*1 and p.Gly583*) newly confirmed to be pathogenic using a NF-κB reporter gene activity assay (see the method of NF-κB reporter gene activity assay an **Figure S2** in the electronic supplemental material). All patients had elevated ISs (**Figure 1**). Intriguingly, when the samples were collected, seven patients were assessed as clinically inactive by the attending physician and the patients reported very few symptoms between hospital visits (**Table S2**).

Two out of the three patients with idiopathic pulmonary hemosiderosis (IPH) also demonstrated an upregulation of type I IFN signaling (**Table S3**), despite being in treatment-induced remission for several years. Genetic analyses ruled out COPA syndrome, which is known to be associated with an elevated type I IFN signature and alveolar hemorrhage (33) in two of the patients. Interestingly, one patient with subsequent development of anti-citrullinated protein (ACPA)-positive RA did not have enhanced IFN signaling, while two patients without any autoimmune-related manifestations, other than pulmonary symptoms, demonstrated high ISs.

One patient with chronic active Epstein-Barr virus infection (CAEBV) exhibited moderately enhanced IFN signaling. Expression of the type I IFN signature in one patient with hypersensitivity to mosquito bites was upregulated slightly and was not correlated with other clinical symptoms or viral loads. Interestingly, this patient had a normal type I IFN signature at first, despite having a whole blood viral DNA load higher than that of the patient with CAEBV (32000 vs. 2400 copy/μg DNA). This demonstrates that the presentation of a normal type I IFN signature cannot exclusively rule out the presence of a chronic viral infection. (**Table S4**).

### Clinical characteristics of patients with undifferentiated inflammatory diseases that exhibited an elevated type I IFN signature

Of the 37 patients with undifferentiated inflammatory diseases, just over half (19 patients, 51.4%) demonstrated high ISs. A comparison of the clinical features in patients, with and without elevated ISs, is shown in **Table 1**. Disease onset was earlier in the patient group with high ISs. The clinical and laboratory features that were more frequently found in the high IS group include, chilblain (31.6% vs. 0%, p = 0.0197) and transaminitis (elevation of liver enzymes) (36.8% vs. 5.6%, p = 0.0422). Nodular erythema (47.4% vs. 16.7%) and the presence of autoantibodies (36.8% vs. 11.8%) were also observed more frequently in patients with high ISs. Of note, low titer antinuclear antibody (ANA) expression (i.e., 1:40 or 1:80), which is usually considered clinically insignificant, was frequently detected in patients with high ISs. Thus, the presence of all autoantibodies, including low titer ANAs, was observed more frequently in patients with high ISs (68.4% vs. 17.6%, p = 0.0031).

**Figure 2:**
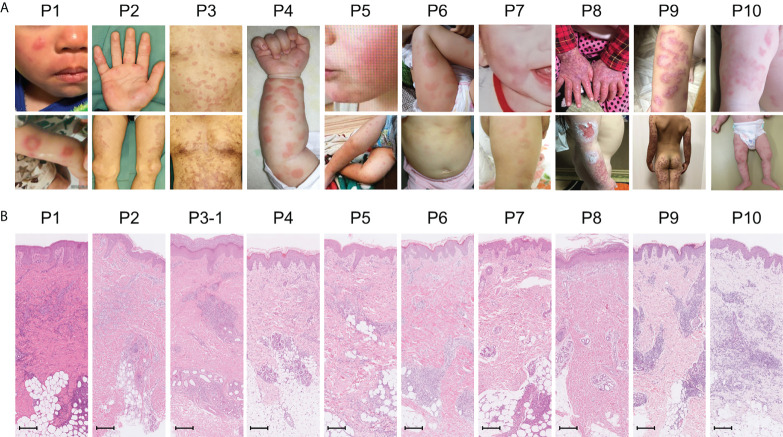
Macroscopic skin manifestations in patients with monogenic interferonopathy-like symptoms. Images of the macroscopic skin manifestations for each patient are shown in panel **(A)**. All patients, with the exception of P8, displayed nodular erythema. These lesions were palpable, sometimes annular, erythematous or violaceous plaques that healed with residual purpura (refer to the lower pictures for P2 and 3). P8 presented with livedo reticularis and skin ulcers. Panel **(B)** shows H&E-stained sections from the skin lesions. In all patients, with the exception of P7 and P8, mononuclear infiltrates in the perivascular and periadnexal dermis were seen. The mononuclear infiltrates in P7 were more intense in deep adipose tissues. Epidermal and superficial dermal infiltrates were observed in P8. The scale bar shown represents 200 μm.

Amongst the 19 patients with high ISs, ten very early onset cases (< 2 years old, **Table 2**, P1-10) presented with some symptoms that led to the suspicion they had monogenic type I interferonopathies, namely, nodular erythema, chilblain-like erythema, panniculitis, myositis, basal ganglia calcification, and interstitial pneumoniae (30) (31). With the exception of P8, all of these patients had similar nodular erythema with post-inflammatory hyperpigmentation, which persisted for weeks to months after resolution, half of which were associated with pain (**Figure 2A**). Skin biopsy results for all ten patients were available and are shown in **Figure 2B**. The H&E-stained sections for these patients, with the exception of P7 and P8, showed similar features, consisting of perivascular and periadnexal mononuclear dermal infiltrates with variable positivity for MPO, CD163, and CD3 expression. Most of the MPO-positive infiltrates lacked nuclear segmentation and showed faint CD15 expression, which is usually expressed by mature neutrophils (**Figure S3** and **Table S5**). These findings resembled skin manifestations seen in PRAAS (36). Patient P3 had two available biopsy results; one specimen (3–1) was taken from a red papule on an upper limb, and the other (3–2) was taken from a painful nodular erythema on a lower limb two years after the first biopsy. Although the immunohistochemistry results were limited, the H&E-stained sections of sample 3-1 shared similar characteristics with the other patients, except for P7 and P8. However, sample 3-2 showed more severe inflammation with dermal infiltration of matured neutrophils, leukocytoclastic vasculitis and septal panniculitis (**Figure S3**). The microscopic features of the skin specimens from P7 and P8 resembled those seen in SLE (**Figure S3**). The H&E sample for patient P7 exhibited lobular panniculitis with MPO+/CD15- mononuclear cell infiltration, in addition to superficial and deep perivascular dermatitis with interface vacuolar degeneration. Patient P8’s sample showed superficial, dermal CD3-positive T cell infiltration and vacuolar degeneration of the basal layer. MPO+/CD15- mononuclear cells were not identified in P8. These ten early onset patients were strongly suspected of having monogenic type I interferonopathy. Indeed, one patient was found to have compound heterozygous mutations in *PSMB8*, which were proven to be pathogenic, and the other four patients were found to have rare variants, of unknown significance, in genes linked to type I IFN signaling using a trio-based WES functional assay (manuscript in preparation).

**Table 2:** Genotypes and clinical phenotypes of patients with high ISs.

Patient	Gender	Age at IS analysis	Disease-onset age	IS	Clinical manifestations							CNS lesion	Autoantibody	Number of interferonopathy-like symptoms^a^	Genotype	Treatment	Efficacy
										Recurrent fever	Chilblain							Nodular erythema	Panniculitis	Myositis	Transaminitis	Other symptoms					
**1**	M	1y3m	7m	81.1/39.4/27.7/52.8/46.3	+	+	+	–	+	+	None	−	Anti-DNA Anti-ssDNA Anti-dsDNA	3	Compound heterozygous mutationsin *PSMB8*	1. PSL 2. Baricitinib	1. Partial 2. Effective
**2^b^**	M	6y	11m	32.9	+	+	+	–	−	−	None	−	−	3	Possibly pathogenic variant found in WES	Topical TAC	N/D
**3^b^**	M	42y	Early childhood	53.3	−	−	+	+	−	−	None	−	ANA 1:40	2	Possibly pathogenic variant found in WES	Topical steroid	N/D
**4**	F	3m	At birth	20.1	−	−	+	–	−	−	Hepatosplenomegaly, anemia	−	ANA 1:40	1	Possibly pathogenic variant found in WES	No treatment	−
**5**	M	4y	6m	13/2.6/11.9	+	−	+	–	−	−	None	−	−	1	No known pathogenic mutations found in WES	No treatment	−
**6**	F	1y6m	10d	34.4	+	+	+	–	+	+	None	−	ANA 1:40	3	Possibly pathogenic variant found in WES	Topical steroid	partial
**7^c^**	F	16y	3m	23.3	+	+	+	+	−	+	Arthritis, iritis	Basal ganglia calcification	−	4	No known pathogenic mutations found in WES	1.PSL, 2.CyA 3.MTX, 4.AZA 5.Adalimumab 6.Tocilizumab	1,3,4,5. Partial 2.effective 6.ineffective
**8**	F	7y	1y	72.9/45.2	+	+	−	–	−	+	Livedo reticularis, ulcers, partial necrosis of liver, recurrent pneumoniae, arthritis	−	Anti-cardiolipin Anti-smooth muscle	1	No known pathogenic mutations found in WES	Colchicine	Partial
**9**	M	4y	10d	22.7/90.3	−	+	+	–	−	+	None	−	Anti-smooth muscle	2	No known pathogenic mutations found in TS	Topical steroid	Partial
**10**	F	1y10m	1m	9.5	+	−	+	–	−	−	Interstitial pneumoniae, fever	−	−	1	No known pathogenic mutations found in TS	1.PSL 2.hydroxychloroquine	Both were effective to IP
**11^c^**	F	7y	6y4m	45.7	+	−	−	−	−	+	Thrombocytopenia, anemia, anasarca, rash, parotitis, hepatosplenomegaly, hypocomplementemia, acute renal insufficiency	White matter hyperintensity	ANA 1:160 Anti-SS-A Anti-SS-B	0	No known pathogenic mutations found in TS	1.mPSL pulse 2.IVCY, 3.AZA 4.MMF, 5.Rituximab	Partial
**12**	F	15y	Early childhood	35.9	−	−	−	−	−	−	Anemia, polyclonal hypergammaglobulinemia, systemic lymphadenopathy, hepatosplenomegaly	N/D	ANA 1:80 Anti-cardiolipin Anti-ssDNA RF	0	No known pathogenic mutations found in TS	Tocilizumab	Effective
**13^d^**	F	44y	41y	41/ 11.3/ 33.7	+	−	−	−	−	−	Headache, abdominal pain, arthralgia, weight loss, refractory asthma	−	Anti-sm	0	No known pathogenic mutations found in WES	1.PSL (for asthma) 2.Tofacitinib	1.Ineffective 2.Partial
**14^d^**	F	44y	41y	21.4	+	−	−	−	−	−	Recurrent pneumoniae, persistent cough	N/D	ANA 1:40	0	No known pathogenic mutations found in WES	No Treatment	−
**15**	F	18y	14y	53.3/23.4	+	−	−	−	−	−	Myalgia, conjunctivitis, pharyngitis, abdominal pain, lymphadenopathy, pleural effusion, lytic lesion in a thoracic vertebra with soft tissue swelling.	−	ANA 1:40	0	No known pathogenic mutations found in TS	PSL	Effective
**16**	M	1y10m	8m	37.6/15.4	−	−	−	−	−	+	Craniosynostosis, elongation of APTT	−	ANA 1:40 Anti-dsDNA Anti-cardiolipin	0	No known pathogenic mutations found in TS	No Treatment	−
**17**	F	22y	13y	13.3/ 6.0 10.0	+	−	−	−	−	−	None	−	ANA 1:40	0	No known pathogenic mutations found in TS	Colchicine	Ineffective
**18**	M	6y	4m	9.2/ 1.1	+	−	−	−	−	−	Cold-induced urticaria associated with fever and arthritis, recurrent conjunctivitis	N/D	−	0	No known pathogenic mutations found in WES	Antihistamine	Ineffective
**19**	F	14y	6y4m	5.2	+	−	−	−	−	−	Headache without meningitis, optic disc swelling	−	−	0	No known pathogenic mutations found in TS	No Treatment	−

a: Interferonopathy-like symptoms include nodular erythema, chilblain-like erythema, panniculitis, myositis, basal ganglia calcification, and interstitial pneumoniae. b: Patient 3 is the father of patient 2. c: Patient 7 and 11 have been previously studied (34, 35). d, Patient 13 is an elder sister of patient 14.

M, male; F, female; TAC, tacrolimus; CyA, cyclosporine; MTX, methotrexate; AZA, azathioprine; mPSL, methylprednisolone; IVCY, intravenous cyclophosphamide; MMF, mycophenolate mofetil ; N/D, no data.

The other nine patients with high ISs, but relatively late disease onset, displayed heterogenous clinical phenotypes (**Table 2**, P11-19). Patients 11 and 12 had similar clinical features to TAFRO syndrome and idiopathic multicentric Castleman’s disease – not otherwise specified (iMCD-NOS), respectively, although their diagnosis was not established since their histopathologic findings were not typical of these diseases. Patient 11 could have been diagnosed with SLE under the 2019 EULAR/ACR classification criteria; however, the patient’s symptoms were resistant to intensive immunosuppressive treatments targeting SLE, and the patient subsequently died from massive gastrointestinal bleeding at the age of 9 (34). The other seven patients remained clinically and genetically undiagnosed. Three of these patients (P13, 14 and 16) had robust family histories with autoimmune diseases, indicating a highly susceptible genetic background towards autoimmunity or an enhanced IFN response. Also of note, three patients (P13, 14, and 17) suffered from recurrent fever and various accompanying symptoms; however, aside from their high ISs, no other obvious abnormalities were detected. This was despite a thorough work up for fever of unknown origin, and included several imaging studies (CT, MRI, PET-CT), gastrointestinal endoscopy, and bone marrow examination. Interestingly, these three patients showed no significant elevation of inflammatory reactants, such as C reactive protein (CRP) and erythrocyte sedimentation rate (ESR), even during febrile episodes; yet, they continued to show constitutively elevated levels of ISs even without apparent symptoms.

### The therapeutic effect of a JAK inhibitor on a pediatric case of PRAAS

**Figure 3:**
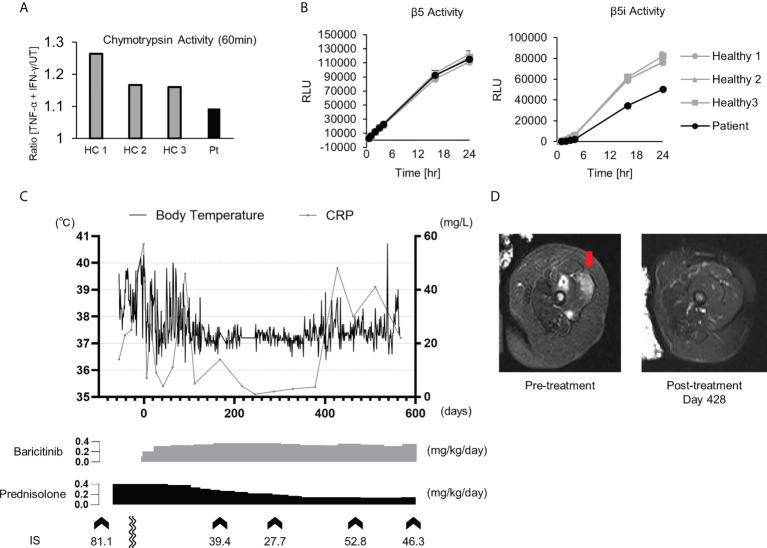
Imaging findings, proteasome activity and clinical course after baricitinib application in P1. **(A)** Ratio of the chymotrypsin-like activity in monocytes with and without stimulation by TNF-α and IFN-γ. The upregulation of chymotrypsin-like activity in monocytes from the patient (Pt) was weaker when compared to healthy controls (HCs) upon induction of the immunoproteasome assembly by IFN-γ and TNF-α. **(B)** Depicts the chymotrypsin-like proteolytic activity of the constitutive proteasome (β5 subunit) and the immunoproteasome (β5i subunit). **(C)** Transitive graph showing the daily maximum body temperature, C reactive protein levels, and the intermittently measured interferon scores of the patient. **(D)** A T2-weighted axial MRI depicting high intensity in the femoral muscle before (left) and after (right) treatment with baricitinib.

Patient 1 in **Table 2** suffered from a cyclic fever and chilblains-like erythema on the extremities and cheeks, both of which started in early infancy. TS analysis identified a previously reported pathogenic variant (c602G>T, p.Gly201Val) and a novel frame-shift variant (c.389delT, p.129Argfs*27) within the PSMB8 gene. Compound heterozygosity was confirmed by Sanger sequencing in the patient’s parents. Analysis of the cDNA derived from the patient’s whole blood cells indicated that the mRNA containing the c.389delT variant was eliminated probably by nonsense-mediated mRNA decay. Moreover, decreased catalytic activity of the immunoproteasome subunit β5i in monocytes of the patient compared with healthy controls confirmed the pathogenicity of the novel frameshift variant (**Figures 3A, B**). These results in addition to the patient’s clinical manifestations and elevated type I IFN signature confirmed a diagnosis of PRAAS.

Although oral prednisolone (PSL; 1 mg/kg/day) quickly induced remission, the dosage could not be reduced below 0.5 mg/kg/day due to relapse. As the patient became steroid-dependent, baricitinib, a selective JAK1/2 inhibitor, was administered. Oral baricitinib was started at 0.1 mg/kg/day and titrated to 0.38 mg/kg/day. The patient’s spiking fever and myositis resolved and CRP levels decreased to a normal level, corresponding with the dose escalation (**Figures 3C, D**). The patient’s daily PSL dose was reduced from 0.4 mg/kg to 0.15 mg/kg. However, discontinuation of the PSL was difficult due to a relapse of occasional low-grade fever and mild erythema, despite that the patient was treated with a higher dose of baricitinib than a typical therapeutic dose for RA (**Figure 3C**) (37).

## Discussion

This study identified several patients with clinically and genetically undifferentiated inflammatory disease that had a demonstrated, enhanced IFN signature. Upregulated IFN signaling was also observed in diseases where the association between the etiology and type I IFN has not been completely established, i.e., clinically quiescent HA20 and IPH.

The relatively large number of clinically and genetically undiagnosed patients with enhanced IFN signatures was surprising. Notably, the clinical and laboratory features found more frequently in patients with high ISs potentially might have resulted from enhanced type I IFN signaling. For example, type I IFN is important for promoting the survival and activation of B cells and thus is involved in tolerance breach and autoantibody production (38). In addition, impaired liver function is a common side effect of IFNβ, which is used for multiple sclerosis treatment (39).

Especially, 10 undiagnosed patients with upregulated type I IFN signaling presented with very early disease onset (an average of 4.5 months) and possessed some clinical characteristics indicative of monogenic type I interferonopathies. Several shared, unique characteristics were observed in the microscopic features of the patients’ nodular erythema, which resembled histological findings in PRAAS. These findings support the possibility that these patients have some Mendelian genetic defects associated with genes related to type I IFN signaling. Consistent with this theory, a causal mutation was identified in the PSMB8 gene of one patient, and four others were found to have rare variants, of unknown significance, in genes that may be associated with type I IFN signaling. Measuring the IFN signature, in these cases, was useful to narrow down the candidate variants found through genetic analysis. In addition, considering the similarities in type I interferonopathy-like clinical manifestations that may be induced by upregulated type I IFN, it is possible that treatment with JAK inhibitors may be effective in patients without confirmed pathogenic mutations. The possibility of utilizing personalized medicine in patients with undifferentiated inflammatory diseases, based on clinical phenotypes and IFN signatures, to identify patients who will respond to treatment with JAK inhibitors, will be important to determine in future studies.

Several patients with undiagnosed inflammatory diseases that had no symptoms indicative of type I interferonopathies were also identified as having enhanced type I IFN signaling, likely for heterogenous reasons. The diseases in two of these patients were clinically, but not histopathologically, compatible with iMCD-TAFRO and iMCD-NOS respectively. MCD clinical manifestations are believed to be driven by excessive expression of proinflammatory cytokines, particularly IL-6; however, the effectiveness of an IL-6 blockade or other immunosuppressants varies between patients, implying that this syndrome is a heterogenous disease (40, 41). Several recent reports have indicated that IFN signaling was upregulated in some patients with TAFRO syndrome (42, 43), and an inhibitor of mTOR, a molecule downstream of type I IFN signaling, was effective (44). Thus, determining whether an IFN signature can be utilized as a biomarker to classify and predict treatment responses in the patients with iMCD is of interest. Some of the patients in the study cohort had a strong family history of autoimmune disease. This is interesting considering the fact that enhanced production of type I IFN has been frequently reported in healthy relatives of SLE patients (45) and in patients with a phase of subclinical autoimmunity (46). Thus, these patients may be at risk for progression to full blown autoimmune disease in future.

Baricitinib was effective for the treatment of PRAAS, but a rather high dose was required to suppress inflammation, as reported previously (37). Interestingly, although the patient’s IS was decreased considerably by a high dosage of baricitinib, it remained abnormally elevated even during the clinically quiescent phase. As the IFN signature is reportedly correlated more strongly with tapering of corticosteroid doses compared with acute-phase reactants such as CRP (23), his symptoms relapsed after reduction of the corticosteroid dose. Although a higher dosage of JAK inhibitors may further suppress IFN signaling and reduce the corticosteroid dose requirement, it could increase the risk of severe adverse effects such as infection and venous thromboembolism (47). Further studies are necessary to determine the optimal dosage of JAK inhibitors for the treatment of type I interferonopathies.

An association between IFN and the etiology of HA20 was first proposed in 2019 (48). Contrary to a previous report (49), an enhanced IFN signature was observed in clinically inactive patients in this study. However, one must consider that these findings are dependent on self-reporting from the patients as well as an assessment of disease activity from the attending physicians, differing from the previous report (49), in which an autoinflammatory disease activity index was used. The results obtained in this study indicate that patients with HA20 may possess a constitutive elevation of ISGs, rather than temporary elevation during flares. It is important from both an etiological and clinical standpoint, to determine whether all patients with HA20 have a constitutive upregulation of type I IFN signaling, as in other monogenic type I interferonopathies. If so, an assessment of the IFN signature in patients will be helpful for diagnosis; for example, when variants of unknown significance are found in the TNFAIP3 gene, as in the present study. A larger study cohort will be necessary to answer this question.

IPH is a rare disorder characterized by diffuse alveolar hemorrhage. Although its etiology remains unknown, the involvement of immunological abnormalities has been suggested based on the presence of autoimmune antibodies (50–52) and the subsequent development of other autoimmune disorders, which have been observed in number of patients with IPH during follow-up (52–55). Some investigators have suggested that circulating immune complexes deposited into the pulmonary capillaries were involved in the disease pathogenesis (50), which may provoke the upregulation of type I IFN signaling. In this study, two out of three patients diagnosed with IPH had elevated IFN signatures, suggesting a possible link between type I IFN and the etiology of IPH. Further assessment of the type I IFN signature in a cohort of IPH patients may help to characterize this heterogenous disease and provide insight into its etiology. In addition, the IFN signaling pathway could provide a potential target for the treatment of this potentially fatal disorder.

There were some limitations in this study. First, the study cohort was recruited based on the recommendation of the attending physician; therefore, patients with clinical findings indicative of interferonopathies were more likely to be recruited. Second, since the clinical information was collected retrospectively, and not all patients were systematically assessed, limited information was available for some patients. For example, three patients in the study cohort had normal IFN signatures and nodular erythema; however, neither skin images nor histological results were available for these patients. Therefore, no comparison could be made with regard to their skin manifestations and the nodular erythema observed in patients with high IFN signatures. Third, ISs were measured only once in 10 of the 19 patients with high IFN signatures. IFN signatures are known to be elevated during infection; while no evidence of infection was observed during blood sampling, it may be more accurate to repeat the assessment in order to rule out a temporary elevation in IS, especially in patients where a moderate elevation of IFN signature was measured. Fourth, 8 of 18 patients with undifferentiated inflammatory diseases and ISs within normal range did not undergo in-depth genetic analysis. Therefore, we cannot conclude that a normal IFN signature can rule out a diagnosis of type I interferonopathy.

Overall, this study demonstrated that a subset of patients exist that have an upregulation of type I IFN signaling without any confirmed disease-causing mutations. Some of these patients may have unknown pathogenic genotypes in genes associated with an upregulation of type I IFN signaling. In some patients, an assessment of the type I IFN signature was useful to narrow down candidate gene variants identified by genetic analysis. The type I IFN signature, in combination with other clinical findings, has the potential to become a useful biomarker for disease diagnosis and treatment choice in the care of patients with inflammatory diseases, although further longitudinal and intervention studies are necessary.

## Data availability statement

The original contributions presented in the study are included in the article/**Supplementary Material**. Further inquiries can be directed to the corresponding author.

## Ethics statement

The studies involving human participants were reviewed and approved by the Kyoto University Hospital ethics committee. Written informed consent to participate in this study was provided by the participants’ legal guardian/next of kin. Written informed consent was obtained from the individual(s), and minor(s)' legal guardian/next of kin, for the publication of any potentially identifiable images or data included in this article.

## Author contributions

TakM, YH, and KIz designed the experiments and authored the manuscript. The first draft of the manuscript was written by TakM. TakM and YH performed the experiments, and TakM analyzed the data. TakM, YH, KIz, NKan., HO, TS, YNa, SA, KM, MB, YNi, AI, TF, DN, NI, YOt, SI, MN, KT, TN, TU, YOh, YT, MS, TE, KIw, AK, TKaw, TadM, TT, SO, and TY collected the clinical data and provided samples (from patients and relatives) for the analyses. MI-N, HN, JA, EH, JT, RN, and TY provided critical conceptual input and helped author the manuscript. SK and HO performed the NF-kB reporter gene activity assay. NKas and MKS analyzed proteasome activity. MF, TatM, and TKan prepared the microscope slides. MF and NKam performed the microscopic analysis of the skin samples. OO conducted the genetic analysis. All authors contributed to the article and approved the submitted version.

## Funding

This research was supported by the following grants: 1. A Health Labor Sciences Research Grant for Research on Intractable Diseases from the Ministry of Health, Labor and Welfare (MHLW) of Japan (H29-Nanchi-Ippan-020 and JPMH20317089 to KI, NK, TY, and RN), 2. Grants-in-Aids for Young Scientists (grant JP19K17293 to KI, JP20K16889 to TS, and JP20K16924 to YH), 3. Grants-in-Aids for Scientific Research (C) (grant JP19K08320 to TT), 4. Grants-in-Aids for Scientific Research (B) (grant 19H03620 to SO), 5. Grants-in Aids for Scientific Research (C) (grant JP19K08798 to NK), 6. Grants-in-Aids for Scientific Research (C) (grant JP22K07867 to KI), 7. the Practical Research Project for Rare/Intractable Diseases from the Japan Agency for Medical Research and Development (AMED) (JP19ek0109200 and JP20ek0109477 to KI, 20ek0109387 to KI and RN, JP20ek0109480 to SO), 8. a research grant from the Morinaga Hoshikai to KI, the Core Center for iPS Cell Research of Research Center Network for Realization of Regenerative Medicine from AMED [JP21bm0104001 to MKS], 9. and the Acceleration Program for Intractable Diseases Research utilizing Disease-specific iPS cells from AMED [17935423 to MKS].

## Acknowledgments

The authors would like to thank Kumi Kodama for technical assistance, Kazuyoshi Kubo, Takatoshi Tsuchihashi, and Takashi Ishikawa for providing the patients’ clinical information, the Center for Anatomical, Pathological and Forensic Medical Research and the Kyoto University Graduate School of Medicine for preparing the microscope slides, and JAM POST for English language editing assistance.

## Conflict of interest

The authors declare that the research was conducted in the absence of any commercial or financial relationships that could be construed as a potential conflict of interest.

## Publisher’s note

All claims expressed in this article are solely those of the authors and do not necessarily represent those of their affiliated organizations, or those of the publisher, the editors and the reviewers. Any product that may be evaluated in this article, or claim that may be made by its manufacturer, is not guaranteed or endorsed by the publisher.
