# Supplementary material for: Assessment of type I interferon signatures in undifferentiated inflammatory diseases: A Japanese multicenter experience
Source: Front Immunol. 2022 Sep 23;13:905960. doi: 10.3389/fimmu.2022.905960 (PMC9541620; doi:10.3389/fimmu.2022.905960)
Supplement: Supplementary file 1 [file DataSheet_1.pdf]

Supplementary Material

1 Supplementary Data

Nuclear factor-kB (NF-κB) reporter gene activity assay

NF-κB reporter gene activity was measured as previously reported[30]. HEK293T cells, in 96-well plates, were transfected with 10 ng per well of the following vectors: a pcDNA3.1+ mock vector, pcDNA3.1+ containing WT myc-A20, or pcDNA3.1+ containing myc-A20 variants. DNA transfection was carried out using Lipofectamine 2000 (Invitrogen, Carlsbad, CA) according to the manufacturer's instructions. The NF-κB luciferase reporter construct and a *Renilla* luciferase control were co-transfected into the cells. After transfection, cells were incubated for 24 hours, and then stimulated with 20 ng/mL tumor necrosis factor α (TNF-α) (R&D Systems, Inc., Minneapolis, MN) for 6 hours. Luciferase reporter activity was analyzed using the Dual-Luciferase Reporter Assay System (Promega, Madison, WI). The activity values for WT and each variant were normalized to that of the mock vector transfection, stimulated with 20 ng/mL TNF-α.

2 Supplementary Figures and Tables

Table S1: Details of the numbers of patients and samples from each disease group.

	Number of patients	Number of samples	Details
Healthy controls	11	11	
Pediatric controls	12	12	
AGS1	2	2	
AGS5	1	1	
AGS7	2	2	
SAVI	3	3	
COPA	2	2	
PRAAS	4	5	1 patient was evaluated twice.
SPENCDI	2	2	
SLE	2	2	
DLE	1	1	
DM	7	7	
CGD	2	3	1 patient was evaluated twice.
Early-onset Behcet's disease	1	1	
HA20	9	9	
IPH	3	5	2 patients were evaluated twice.
CAEBV	1	1	
HMB	1	3	1 patient was evaluated three times.
Kawasaki disease	1	1	
Takayasu arthritis	2	2	
CAPS	2	2	
FMF	2	4	2 patients were evaluated twice.
PAAND	1	1	
PFAPA	6	6	
Undiagnosed	37	52	Patients with a low IS were evaluated once. Some patients with a high IS were evaluated multiple times, details of which are described in Table 2.
Total	117	140	

Table S2: Genotypes and clinical phenotypes of patients with A 20 haploinsufficiency.

Patient	Gender	Age at IS analysis	Disease-onset age	IS	genotype	Initial diagnosis before genetic analysis	CRP at IS analysis (mg/L)	Symptoms at IS analysis	Treatment at IS analysis
1	F	37y	Infancy	40.3	c.986+1 G>T, p.Lys329Asn*1 hetero	SLE (Recurrent fever, lymphadenitis, arthritis)	N/D	None	PSL, Hydroxychloroquine
2	M	15y	4y	41.0	c.133C>T, p.Arg45* hetero	Crohn's disease	N/D	None	Colchicine, Mesalamin
3	F	6y	7m	51.7	c.1747G>T, p.Gly583* hetero	Recurrent fever with arthralgia and abdominal pain	7	None	No treatment
4	M	10y	1y1m	48.2	c.133C>T, p.Arg45* hetero	PFAPA-like recurrent fever → intestinal Behçet's disease	5	Stomatitis	Colchicine
5[a]	F	5y	6m	46.2	c.2209delC, p.Gln737Serfs*79 hetero	FMF like autoinflammatory disease	<0.2	None	Etanercept
6[a]	F	29y	5y	56.0	c.2209delC, p.Gln737Serfs*79 hetero	Crohn's disease, Hashimoto's disease	7.1	None	No treatment
7[a]	F	68y	Early childhood	44.3	c.2209delC, p.Gln737Serfs*79 hetero	Recurrent stomatitis, Hashimoto's disease	N/D	None	No treatment
8	F	19y	11y	27.1	c.252delC, p.Trp85Glyfs*11 hetero	RF negative pJIA → intestinal BD	0.8	None	ADA, PSL, Celecoxib, Iguratimod
9	M	4y	1y	24.7	c.2088+5G>C, p.His636Glufs*55 hetero	sJIA → Psoriatic arthritis	11.3	Skin erythema, adrenal insufficiency	IFX, MTX, PSL, Naproxen, Colchicine

a: Patients 6 and 7 are the mother and grandmother of patient 5, respectively.

M: male, F: female, PSL: prednisolone, ADA: adalimumab, IFX: infliximab, MTX: methotrexate

Table S3: Clinical phenotypes of patients with idiopathic pulmonary hemorrhage.

Patient	Gender	Age at IS analysis	Disease-onset age	IS	Clinical manifestation at diagnosis				Extrapulmonary manifestations	Symptoms at IS analysis	Auto-antibodies	Hemosiderin-laden macrophages	Treatment	Genotype
					Anemia	Cough	Dyspnea	Hemoptysis						
**1**	F	15y	4y	93.7/ 48.6	+	+	+	+	None	None	ANA 1:320, Anti-ssDNA, Anti-dsDNA, Anti-SS-A	+ (gastric aspirate)	PSL, AZA, ICS, CAM	No pathogenic mutations found in the NGS-based gene panel test
**2**	F	13y	4y	11.2/ 13.5	+	+	−	+	None	None	ANA 1:80	+ (sputum)	PSL, ICS, CAM	Not analyzed
**3**	F	20y	2w	1.62	+	+	+	+	Arthralgia, morning stiffness, annular erythema	None	ACPA, RF	N/D	Iguratimod (for arthralgia)	No mutations in the *COPA* gene

ACPA: anti-cyclic citrullinated peptide antibody, AZA: azathioprine, ICS: inhaled corticosteroid, CAM: clarithromycin, NGS: next-generation sequencing

Table S4: Clinical phenotypes of patients with chronic active Epstein-Barr virus disease (CAEBV) and hypersensitivity to mosquito bites (HMB).

Patient	Gender	Age at IS analysis	Disease-onset age	IS	diagnosis	Clinical manifestation at sampling				EBV DNA load in whole blood (copy/µgDNA)	Infected cell type	Treatment
						fever	Skin rash	lymphadenopathy	hepatosplenomegaly			
**1**	F	1y4m	1y1m	12.2	CAEBV	+	+	+	+	2400	T cell	PSL
**2-1**		3y4m		3.3		−	+	−	−	32000		None
**2-2**	F	3y10m	2y4m	13.6	HMB	−	++	−	−	52000	NK cell	None
**2-3**		5y2m		26.0		−	++	−	−	15000		None

Table S5：A summary of histological and immunohistochemical findings for the 10 samples studied.

**Patient**	**1**	**2**	**3-1**	**3-2**	**4**	**5**	**6**	**7**	**8**	**9**	**10**
**CD163**	++	++	N/D	+++	+++	+++	+++	++	++	++	+++
**MPO**	+++	++	++	+++	+	++	++	+	-	+	++
**CD15**	+	-	N/D	+++	+	+	-	+	-	+	+
**CD123**	+	-	N/D	-	-	-	++	-	-	++	+
**CD3**	+	++	N/D	++	+	++	++	+	++	+++	++
**CD20**	-	+	N/D	+	+	+	+	-	-	+	+
**Dermal infiltrate pattern**	Perivascular and interstitial	Perivascular and interstitial	Perivascular	Perivascular	Perivascular and interstitial	Perivascular	Perivascular and interstitial	Perivascular	Superficial perivascular	Perivascular	Perivascular and interstitial
**Epidermal change**	-	-	-	-	-	-	-	Vacuolar degeneration	Vacuolar degeneration	-	-
**Vasculitis**	-	-	-	+	-	-	-	-	-	-	-
**Panniculitis**	-	-	N/D	Septal	-	-	-	Lobular	-	-	-

N/D: no data

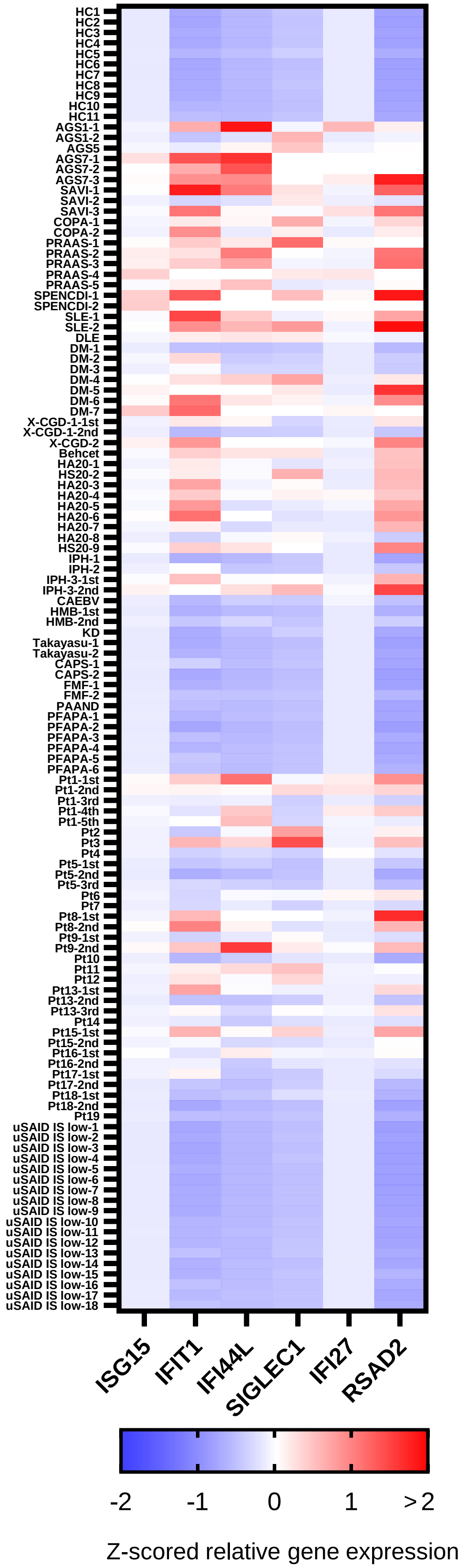

Figure S1- *A heatmap of z-scored relative expression of 6 interferon stimulated genes.* Colums show z-scored relative expression of each interferon stimulated gene. Rows show a diagnosis of each patient. Results of re-examination are expressed by ordinal numbers.

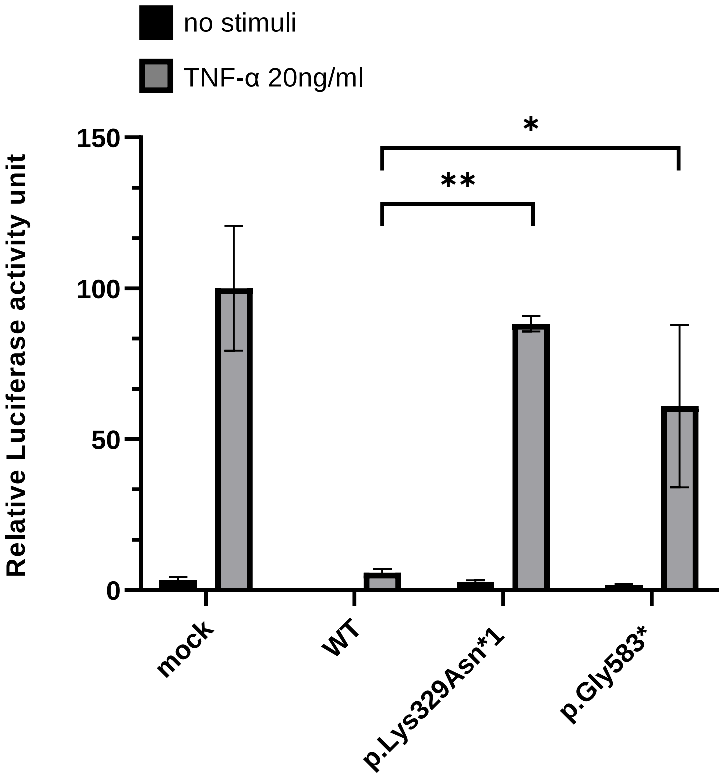

Figure S2- *Results of the NF-κB reporter gene activity assay.* All experiments were performed in triplicate. Values are expressed as the mean ± standard deviation (SD). Statistical significance of the difference between the WT and variants stimulated with TNF-α was measured by luciferase activity and analyzed using a one-way ANOVA with Dunnett's multiple comparisons test. Calculated p values are indicated; $p < 0.05$ with * and $p < 0.001$ with **. Suppression of the TNF-α induced NF-κB activity by the *TNFAIP3* variants was significantly lower than that seen in the WT.

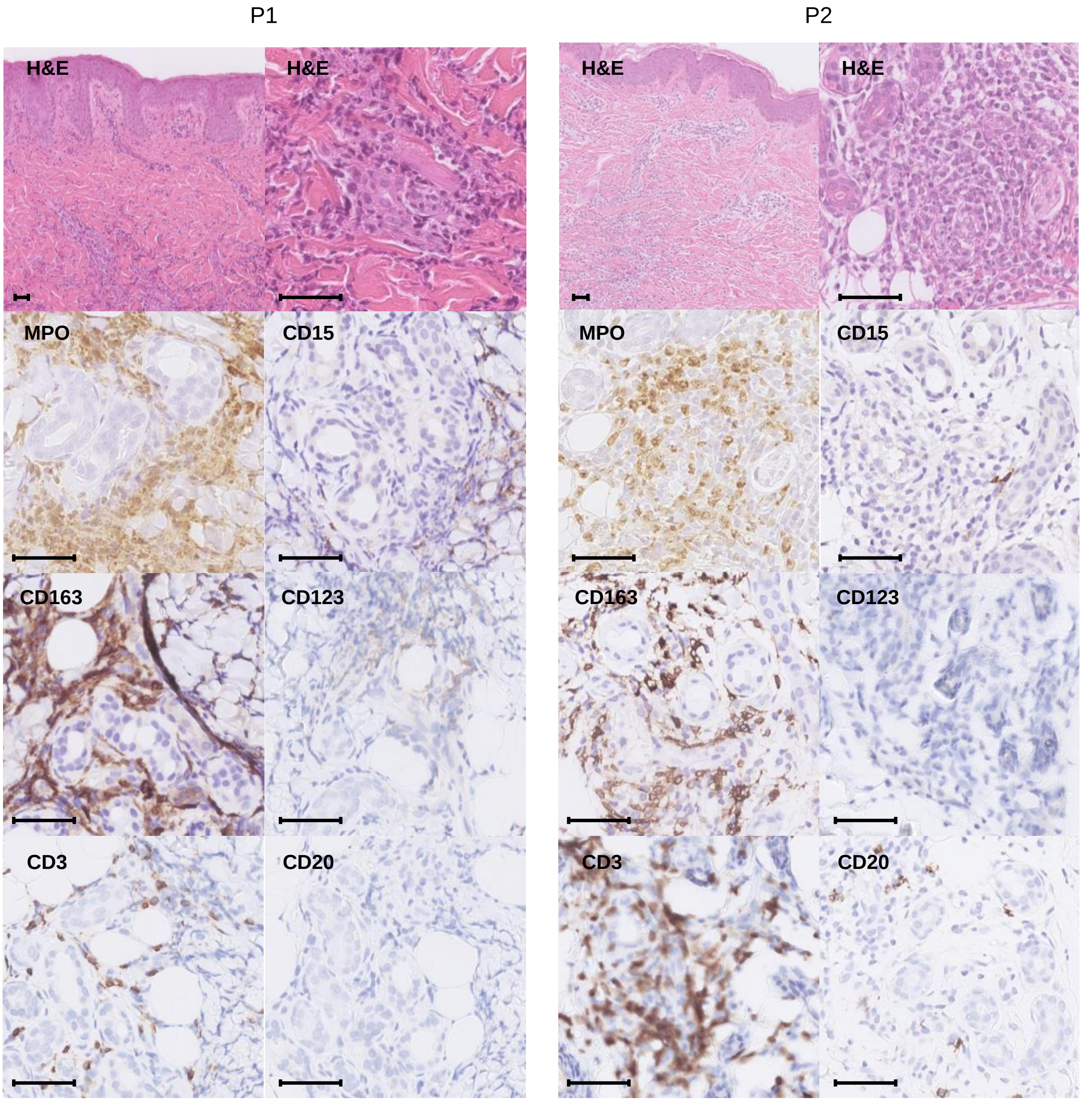
P1
P2
H&E
H&E
H&E
H&E
MPO
CD15
MPO
CD15
CD163
CD123
CD163
CD123
CD3
CD20
CD3
CD20

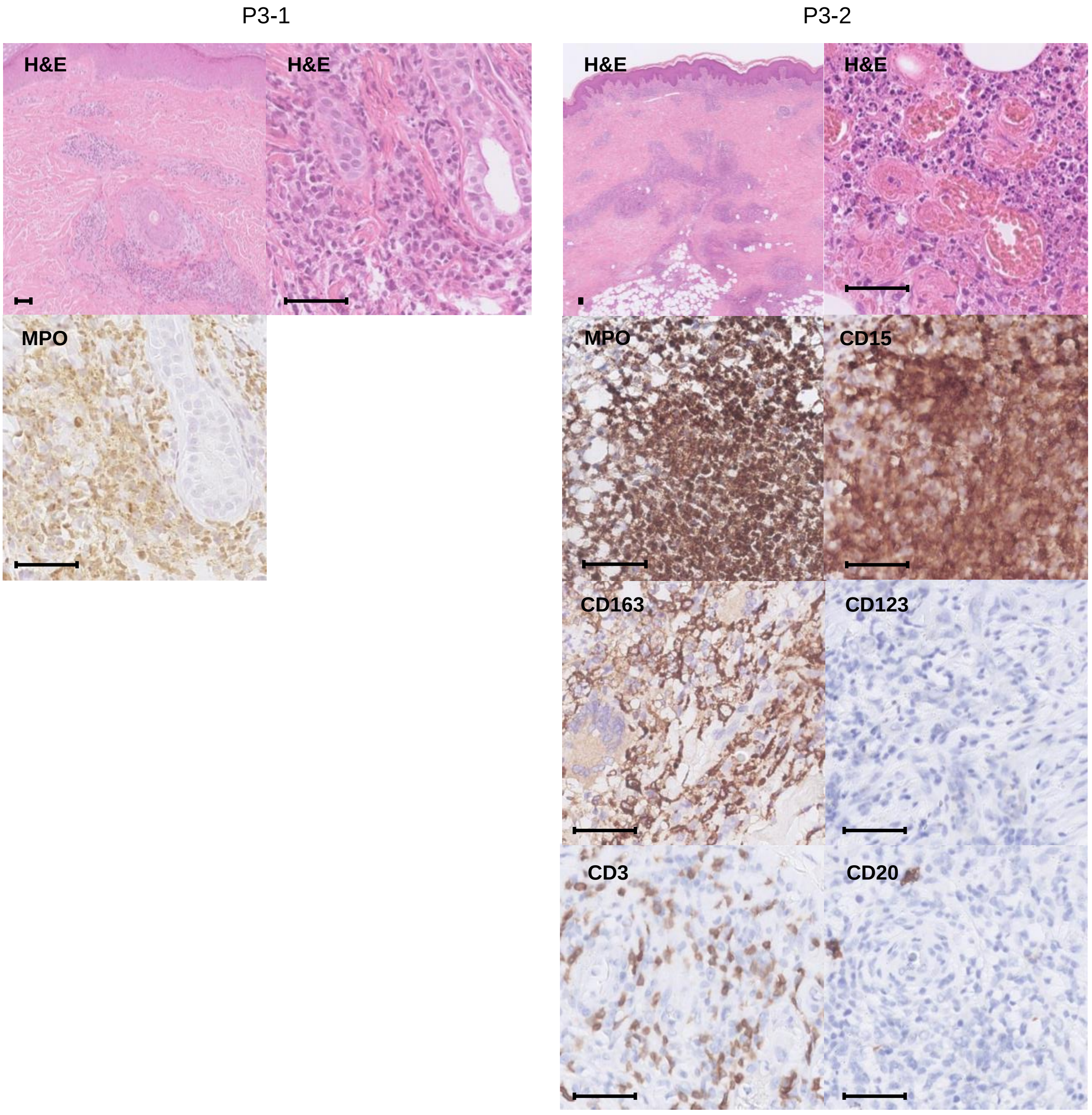
P3-1
P3-2
H&E
H&E
H&E
H&E
MPO
MPO
CD15
CD163
CD123
CD3
CD20

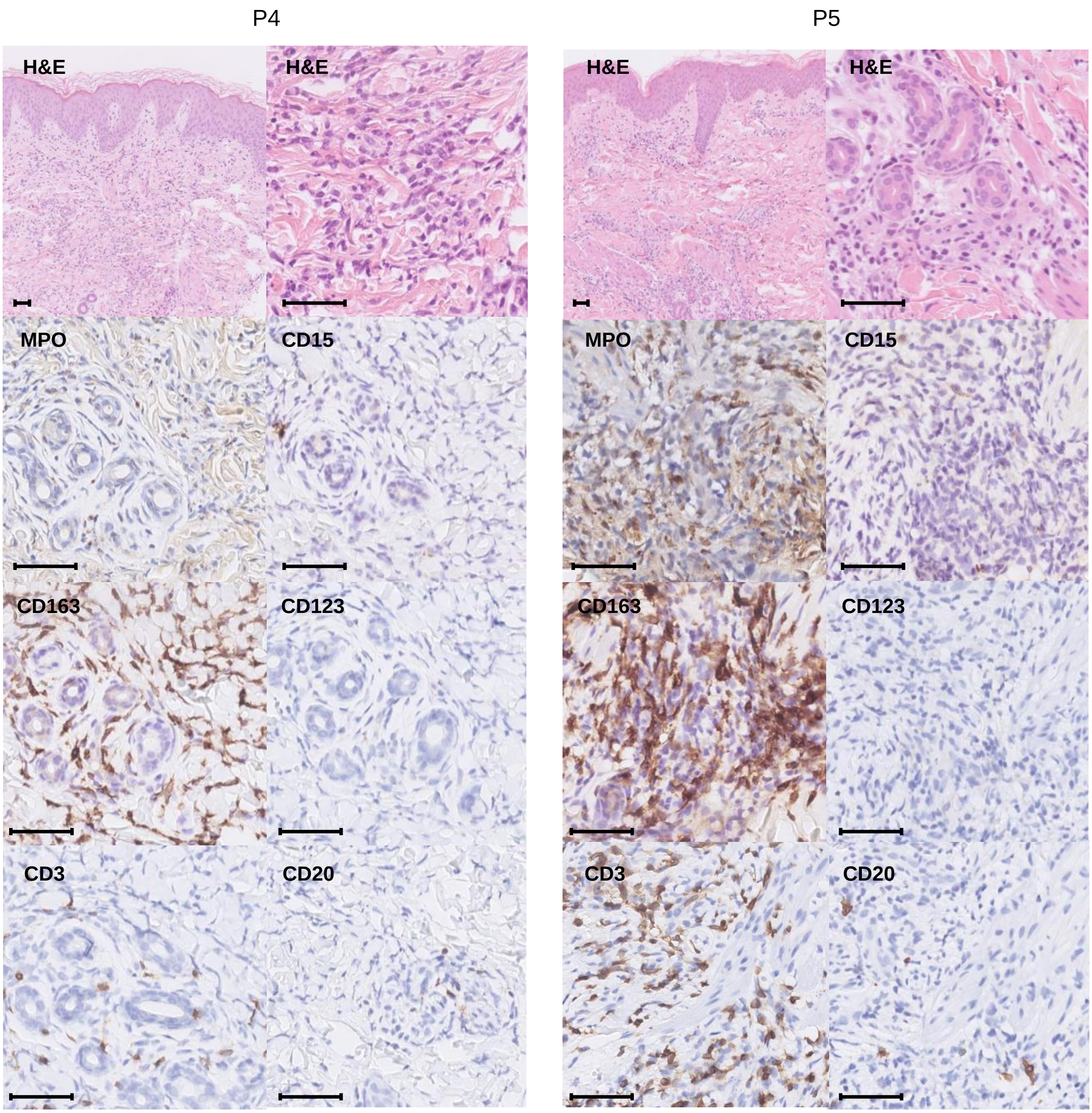
P4
P5
H&E
H&E
H&E
H&E
MPO
CD15
MPO
CD15
CD163
CD123
CD163
CD123
CD3
CD20
CD3
CD20

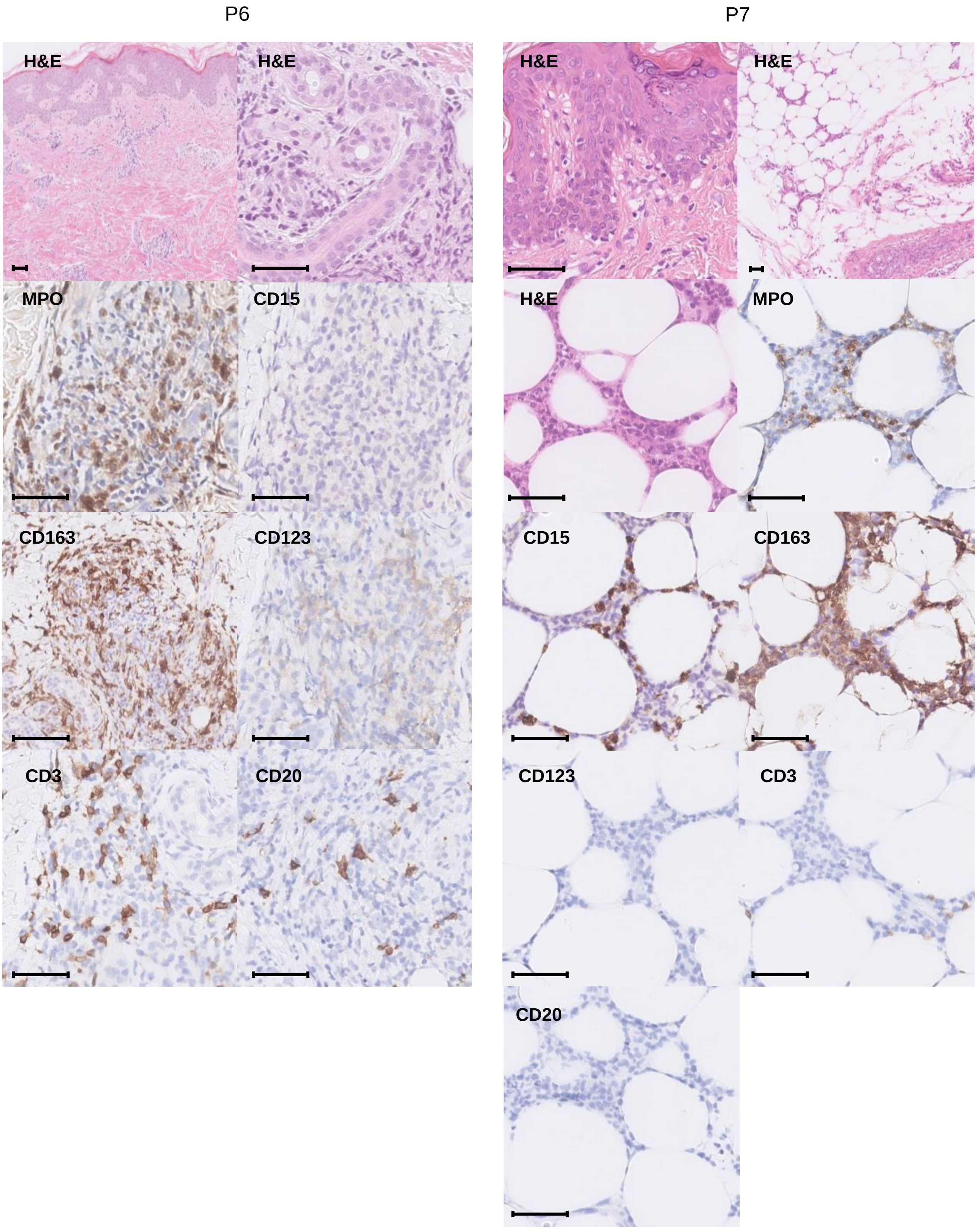
P6
P7
H&E
H&E
H&E
H&E
MPO
CD15
H&E
MPO
CD163
CD123
CD15
CD163
CD3
CD20
CD123
CD3
CD20

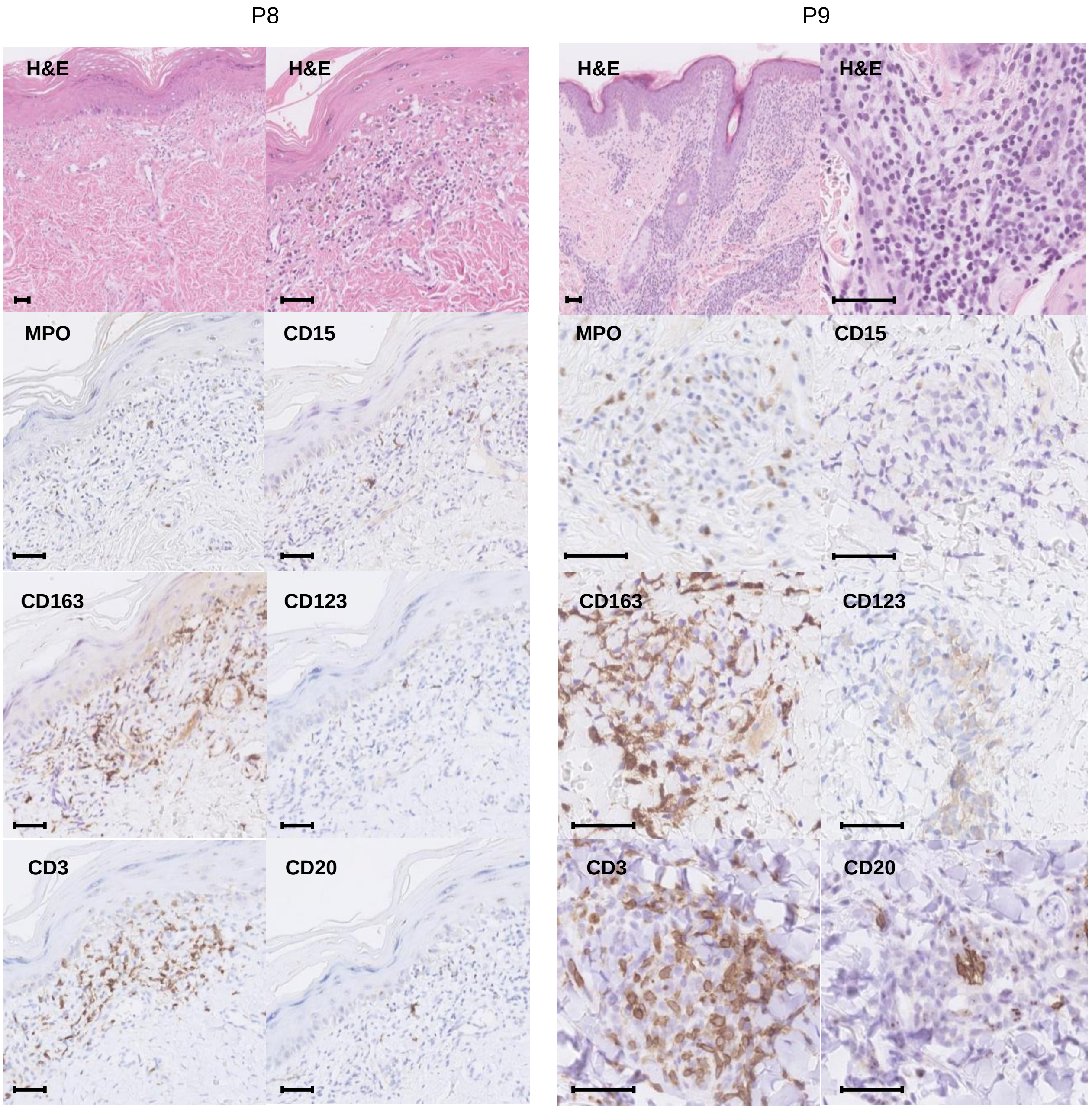
P8
P9
H&E
H&E
H&E
H&E
MPO
CD15
MPO
CD15
CD163
CD123
CD163
CD123
CD3
CD20
CD3
CD20

P10

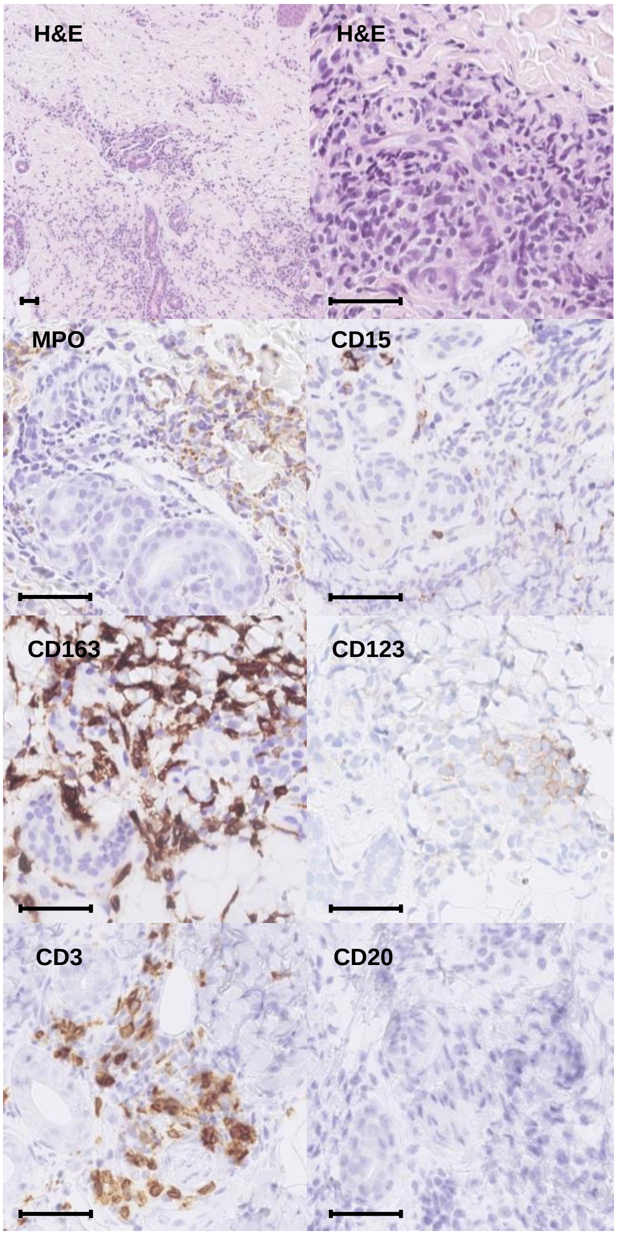

Figure S3- *H&E stained sections of patients.* With the exception of the sections for patients P3-2, P7, and P8, all sections showed superficial and deep perivascular dermal infiltrates without epidermal changes, containing mononuclear cells, many of which were MPO-positive and CD15-negative. The skin specimen for P3-2 showed leukocytoclastic vasculitis and septal panniculitis, in addition to dermal infiltrates consisting of matured neutrophils, histiocytes, and lymphocytes. The H&E stained section for patient 7 showed lobular panniculitis, and superficial and deep perivascular dermatitis with vacuolar changes. Cellular infiltrates mainly consisted of MPO-positive and CD163-positive mononuclear cells. The H&E stained section for patient 8 showed superficial perivascular dermatitis with vacuolar degeneration of the basal layer, and exhibited CD3-positive T cell, but not CD20-positive B cell infiltration. The scale bar for all sections represents 50 μm.
